# Functional Genomics Differentiate Inherent and Environmentally Influenced Traits in Dinoflagellate and Diatom Communities

**DOI:** 10.3390/microorganisms8040567

**Published:** 2020-04-15

**Authors:** Stephanie Elferink, Uwe John, Stefan Neuhaus, Sylke Wohlrab

**Affiliations:** 1 Alfred-Wegener-Institute, Helmholtz Centre for Polar and Marine Research, Am Handelshafen 12, 27570 Bremerhaven, Germany; stefan.neuhaus@awi.de (S.N.); sylke.wohlrab@awi.de (S.W.); 2 Helmholtz Institute for Functional Marine Biodiversity, Ammerländer Heerstraße 231, 26129 Oldenburg, Germany

**Keywords:** arctic, metatranscriptomic, metabarcoding, microplankton, molecular ecology

## Abstract

Dinoflagellates and diatoms are among the most prominent microeukaryotic plankton groups, and they have evolved different functional traits reflecting their roles within ecosystems. However, links between their metabolic processes and functional traits within different environmental contexts warrant further study. The functional biodiversity of dinoflagellates and diatoms was accessed with metatranscriptomics using Pfam protein domains as proxies for functional processes. Despite the overall geographic similarity of functional responses, abiotic (i.e., temperature and salinity; ~800 Pfam domains) and biotic (i.e., taxonomic group; ~1500 Pfam domains) factors influencing particular functional responses were identified. Salinity and temperature were identified as the main drivers of community composition. Higher temperatures were associated with an increase of Pfam domains involved in energy metabolism and a decrease of processes associated with translation and the sulfur cycle. Salinity changes were correlated with the biosynthesis of secondary metabolites (e.g., terpenoids and polyketides) and signal transduction processes, indicating an overall strong effect on the biota. The abundance of dinoflagellates was positively correlated with nitrogen metabolism, vesicular transport and signal transduction, highlighting their link to biotic interactions (more so than diatoms) and suggesting the central role of species interactions in the evolution of dinoflagellates. Diatoms were associated with metabolites (e.g., isoprenoids and carotenoids), as well as lysine degradation, which highlights their ecological role as important primary producers and indicates the physiological importance of these metabolic pathways for diatoms in their natural environment. These approaches and gathered information will support ecological questions concerning the marine ecosystem state and metabolic interactions in the marine environment.

## 1. Introduction

The marine environment covers more than 70% of the world’s surface yet much remains to be described of its structure and function. The ocean is not structured by hard environmental barriers and is considered a highly connected system. Regarding marine microeukaryotes, this has led to a discussion about “everything is everywhere but the environments selects” [1]. However, oceanic provinces have been defined according to Longhurst [2] and confirmed by water chemistry [3] and biodiversity [4] patterns. According to Baas-Becking [1], local environmental processes mediate species diversity and molecular data show regional and global differences in biodiversity and corresponding community structures [5,6,7].

In the context of climate change, species are moving toward polar regions [8] and the biodiversity of planktonic communities is changing; some species may go extinct, whereas new species may be introduced to a community. This can lead to overall diversity loss but can also lead to the same diversity albeit with different compositions. Species counts alone may not represent the overall functional biodiversity of a microeukaryotic community or within an ecosystem. To sustain a stable ecosystem, the preservation of functional biodiversity (with different species) and the occupation of relevant ecological niches may be more important [9,10]. To identify processes of species coexistence and adaptation, species biodiversity has been associated with functional traits of key species [11]. Functional traits can represent different ecological roles and corresponding niches, e.g., nitrogen fixation or motility, and can be compiled into measures of functional diversity [12], thus contributing to an understanding of the biologic community responses to changes in the environment [13,14,15]. For example, arctic organisms must cope with seasonal relatively stable, but low water temperatures, whereas in temperate regions species are exposed to a wider range of water temperatures. Therefore, species require different abilities in trait plasticity to tolerate different environmental parameters.

Previous studies have shown how additional aspects of biodiversity, such as functionality, can be used to predict the functioning and productivity of an ecosystem [9,16,17]. A functional group represents an assembly of species, which are not necessarily taxonomically related yet carry out the same essential functional role. Because they may not be closely related, the species may have diverse constraints (historical, genetic and physiological) in their ability to respond to changing ocean conditions [18]. Measures based on functional traits are strongly coupled to ecosystem structure and functioning [14,19], have been shown to provide useful means of describing plankton community structure and biodiversity [20], and have been used to assess community responses to climate change [21].

Diatoms are one of the most prominent microeukaryotic plankton groups, playing many functional roles, including acting as a primary component of marine autotrophic production and contributing to silica uptake. Diatom species may have some functional overlap with dinoflagellates [13]. Yet dinoflagellates include autotrophic, heterotrophic, parasitic and endosymbiotic species, thus encompassing diverse ecological niches. Differences in the life strategies and nutrition modes of these two groups should be detectable in their expressed genes, as identified for diatoms [22], dinoflagellates [23,24] or both groups [25,26]. However, microeukaryotes are regulated by the interplay of individual species traits, biotic interactions and the environment [15] and these interacting challenges render it difficult to identify specific trophic roles.

Genetic diversity is often assessed with high throughput barcode sequencing, providing substantial information on species biogeography, rare species and patterns of species richness [5,27]. However, barcoding approaches, as well as conventional monitoring with microscopy, lack the resolution to gain sufficient insights into all functional processes. Other molecular approaches, including gene expression analysis, elucidate most cellular and developmental processes of organisms, such as physiological status [28], life stages [29] and cell communication among prey and grazers [30], as well as providing for inference about conspecifics [31]. Most experiments to this end are performed under controlled laboratory conditions and the results can serve as reference data and knowledge for fieldwork employing metatranscriptomic approaches. The detailed knowledge generated in the laboratory allows for the transfer of gene expression analyses into field studies, e.g., to assess more complex community structures [32] and reveal aspects of global biogeography of eukaryotic genes [33]. Metatranscriptomic studies have already contributed significantly to our understanding of the biology of diatoms and dinoflagellates—from genes to ecosystems—and have provided other new insights into niche partitioning and resource use of diatoms [22,34], the impact of abiotic factors on community structure [26,35,36], mechanisms potentially governing diatom and dinoflagellate dominance [25], size-dependency of plastic and conserved community traits of dinoflagellates [23] and the tight circadian-regulated metabolic coupling within marine microbial communities [37].

To improve our understanding of the interplay between taxonomic and functional diversity of a community relative to environmental influence, this study distinguishes functional processes that may be affected by changing environmental conditions from those functions that are affected by biodiversity (species identity). The overall aim of this study is to link the biodiversity and functioning of microeukaryotic communities. In particular, it focuses on: (1) the influence of environmental parameters, i.e., temperature, salinity and inorganic macronutrients, on the functional biodiversity of microeukaryotic communities in arctic and subarctic/temperate waters; (2) the identification of functional processes influenced by these environmental parameters; and (3) the identification of functional processes associated with diatoms and dinoflagellates, the latter group of which exhibits more diverse lifestyles and can have different roles in the marine ecosystem (autotrophic, mixotrophic, heterotrophic, symbiotic and parasitic).

## 2. Materials and Methods

### 2.1. Field Sampling for Plankton and Environmental Parameters

**Figure 1:**
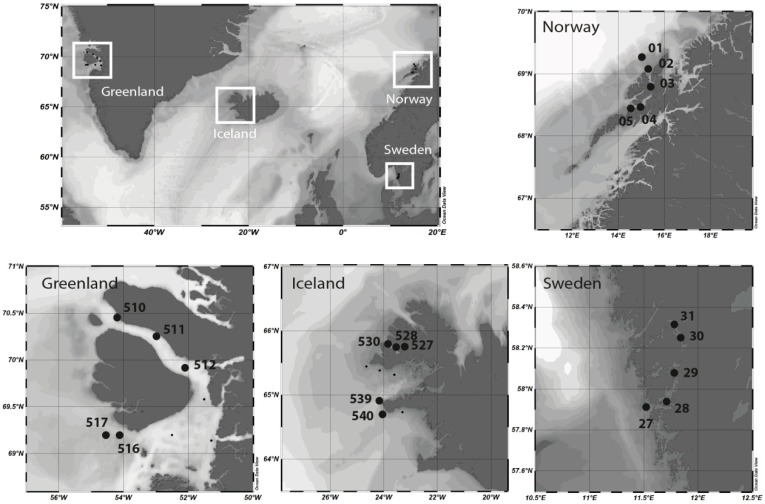
Study sites and sampling stations on the coast of west Greenland, northwest Iceland, Norway and Sweden.

Sampling was performed during the MSM-21/3 ARCHEMHAB expedition [38] aboard the *RV Maria S. Merian* at the western coast of Greenland and northwest Iceland fjord systems and adjacent waters between 27 July and 8 August 2012, and during HE-431 expedition (https://doi.org/10.1594/PANGAEA.863438) aboard the *RV Heincke* at the coast of Norway and Sweden between 23 August and 6 September 2014 (Figure 1). Water samples were collected in the epipelagic zone by Niskin bottles mounted on a rosette-sampler with conductivity, temperature and depth (CTD) sensors to measure temperature and salinity and bio-optical parameters.

Samples for inorganic nutrient measurements (ammonium, nitrate, nitrite, phosphate and silicate) were directly taken from the Niskin bottles, transferred to polypropylene bottles (50 mL) and frozen immediately at −20 °C. Nutrient samples were analyzed with a continuous-flow autoanalyzer (Evolution III, Alliance Instruments, Freilassing, Germany)) based upon standard seawater analytical methods for determination of nitrate and nitrite [39], ammonium [40], silicate [41] and phosphate [42].

*In situ* chlorophyll-*a* concentrations were analyzed by filtering 1.0–2.0 L of seawater through glass microfiber filters (Whatman GF/F, Whatman, Maidstone, UK; nominal pore size: 0.7 µm). The filtration unit was covered to exclude light, and sample filters were frozen immediately at −80 °C until laboratory analysis. Pigments were extracted by sonication of filters in 10 mL 90% acetone and then incubated overnight in darkness at 4 °C. The extract was centrifuged at 3020× *g* for 10 min, and the fluorescence of the supernatant was determined at 665 nm (TD-700 fluorometer, Turner Designs, Sunnyvale, USA). Extracted chlorophyll-*a* data are archived in the data repository PANGAEA [43].

Seawater for DNA extraction was integratively sampled from the surface (1 to 30 m) through a hose connected to a shipboard membrane pump (QBY-25-SS; AGB-Pumpen, Hamburg, Germany). In total, 60 L of seawater per sampling station was passed over a sequential filter tower with progressively decreasing nylon mesh-sizes (200, 50 and 20 µm) to obtain distinct plankton size-fractions. For this study, the microplankton (20–50 µm) size fraction was processed.

Retentates from the filter tower and membrane filters for molecular analyses were back-washed into centrifuge tubes with 0.2 µm-filtered seawater, adjusted to a total volume of 45 mL and split into three 15 mL aliquots. Cell suspensions were centrifuged for 15 min at 3600× *g* at 4 °C, after which the pellet aliquots were separated for subsequent DNA and RNA analysis. Pellets for DNA analysis were resuspended in a 400 µL AP1 lysis buffer (Qiagen, Hilden, Germany), whereas pellets for RNA analysis were resuspended in Tri Reagent (Sigma Aldrich, Darmstadt, Germany) and then each was transferred to a microcentrifuge vial with 0.2 µm glass beads (Sigma Aldrich, Darmstadt, Germany). Vials were shock-frozen in liquid nitrogen and kept at −20 °C (for DNA) or −80 °C (for RNA) until further processing.

### 2.2. Metabarcoding: rRNA Analysis and Taxonomic Assignment

In total, 20 integrated surface plankton samples were sequenced on a 454 FLX+ sequencer. Raw reads were deposited in the European Nucleotide Archive (ENA) via the GFBio portal under Accession No. PRJEB32397 (MSM21-3) and PRJEB37605 (HE-431).

Methodological errors in 454-sequencing and OTU clustering may lead to an over- or underestimation of diversity [44], but recent methodological advances in amplicon sequence denoising promise the capability to reconstruct the true biologic sequences of amplicon surveys [45]. These amplicon sequence variants (ASV) are thus expected to replace the common OTUs as taxa proxies because ASVs resolve single nucleotide differences and provide constant labels across studies [46]. The R package Dada2 [47] was used in this study to obtain such ASVs. Cutadapt [48] was used to remove the leading forward primer from the reads. Reads without detected forward primer sequences were discarded. These pre-processed sequences were further processed with R version 3.5.2 [49] using functions of the Dada2 package (version 1.11.0). Sequences were truncated to a length of 400 bp. Reads shorter than 400 bp, with the expected base error above 5—and those containing any base with a Phred quality score ≤10—were discarded. ASVs were constructed, cleaned of chimeras and taxonomically annotated following the guidelines of the Dada2 online tutorial (https://benjjneb.github.io/dada2/tutorial.html); settings were adapted to the recommendations for 454 data. The Parc version of the Silva 132 LSU dataset was used as reference for taxonomic assignment [50] and the taxonomic labels were adjusted as described in http://blog.mothur.org/2018/01/10/SILVA-v132-reference-files/.

The ASVs annotated as alveolates or stramenopiles were classified more accurately by PhyloAssigner version 6.166 [51] with a phylogenetic placement onto reference trees based on 18S/28S concatenated alignments (doi:10.5281/zenodo.3741720), according to Elferink et al. [27] 

Statistical analyses were run in the R environment version 3.0.2 [49] with the package ‘vegan’ [52] and ‘plyr’ [53]. The sequence numbers per sample were not subsampled to the smallest sequence number of any sample to avoid an increase in the number of potential false positives [54] by comparison to the relative abundance of counts in samples. Rarefaction and ASV (species) accumulation curves of each station were calculated in vegan using the function ‘rarecurve’ and created after quality control and clustering to represent the local species richness and to serve as a reference for the diversity captured in a sample. The final ASV table was Hellinger-transformed (double-square root transformation) as recommended for clustering or ordination of species abundance data [55,56] with the ‘decostand’ function. A principal coordinate analysis (PCoA) was applied to the Bray-Curtis dissimilarities matrix of ASVs. Environmental factors were Hellinger-transformed to normalize the dataset and standardized by the factors’ maximum values. To compare environmental similarities among samples, principle component analysis (PCA) with calculated Euclidean distances was carried out (log10 transformation for normalization). The package ‘treemap’ was used to illustrate the taxonomic hierarchical structure among regions.

### 2.3. Metatranscriptomics: mRNA Analysis and Functional Assignment

All raw Illumina paired-end sequencing reads of 20 reverse-transcribed mRNA samples from the microplankton size-fraction (20–50 µm) were deposited at the European Nucleotide Archive (ENA) via the GFBio portal under Accession No. PRJEB37134 (MSM21-3) and PRJEB37606 (HE-431).

Transcriptomes were trimmed (length > 75 bp), ‘De Novo’ assembled in CLC Genomic Workbench 11 contigs blasted (blastn) against the MMETSP database [57] with BLAST to obtain taxonomic information. Raw reads from the obtained metatranscriptomes were mapped to the consensus sequence to obtain read counts per contig. Subsequently, read counts per contig were normalized with the R package ‘limma’ by applying the normalizeQuantile function [58]. Functional annotation of the contigs was conducted by Trinotate v3.0.2 and predicted from the top blastx hits (e-value ≤ 10^−9^).

Variance partition analyses were done in the R environment with the package ‘variancePartition’ and ‘DESeq.’ The functional responses of temperature, salinity, total nitrogen, phosphate, silicate, chlorophyll-*a*, dinoflagellate abundance and diatom abundance were analyzed. From the variance partition model, the estimates for all parameters were used to calculate from which environmental parameter most influenced the respective Pfam. This was the case when the variance explained by a particular parameter (e.g., temperature) exceeded the variance explained by all other parameters considered, including the residual variance. The significance of one Pfam for a region was identified with permutation (times = 1000, function permatfull from ‘vegan’ package) and filtering (*p* value = 0.05/(17926*20[stations]). To obtain the respective Pfam domains for dinoflagellates and diatoms, two separate variance partition models were conducted (each with the environmental parameter identified above), because of the strong negative correlation between groups in our data.

Further, KEGG Orthologies (KOs) were assigned to Pfam domains and the KO abundances counted because one Pfam domain can result in different KOs and different Pfam domains can result in the same KO. For visualization of the data, results were plotted for each environmental parameter separately with the web application FuncTree2 [59].

## 3. Results and Discussion

This study links the biodiversity and associated functional traits of microeukaryotic communities in arctic and subarctic/temperate waters, with emphasis on dinoflagellates and diatoms. The two taxonomic groups encompass different life strategies and niches within the marine environment, with diatoms being functionally similar, i.e., all silicifying and primarily photoautotrophic, whereas dinoflagellates comprise highly diverse nutritional modes (autotrophic, mixotrophic and heterotrophic) and lifestyles (free-living, symbiotic or parasitic). To improve our understanding of the interplay between biodiversity and functional diversity of a microeukaryotic community relative to environmental influences on the communities, this study distinguishes functional processes that are affected by changing environmental conditions from those functions which are more affected by the species identity. This was accomplished by variance partitioning analysis, i.e., aligning Pfam domain abundances with measured environmental parameters and species abundances. First the community is taxonomically and functionally described to then identify the influence of environmental parameters, i.e., temperature, salinity and inorganic macronutrients, on the diversity and functional diversity of microeukaryotic communities. Second, we identify and discuss the functional processes significantly influenced by these environmental parameters, and finally we then elucidate functional processes significantly influenced within the diatoms and dinoflagellates with their contrasting life strategies and niches.

### 3.1. Taxonomic and Functional Description of the Microplankton Communities

Quality processing of rRNA sequencing reads and the removal of singletons, doubletons and potential fungal and metazoan sequences (defined by dada2) led to an average of 11,714 reads (range: 4565 to 36,266, Table S1, an average of 279 ASVs per sample (range: 29 to 279). Among the total 506 ASVs, 22 ASVs were present in all four regions, and the number of unique ASVs varied across the sampling regions (11 for Sweden to 57 for Iceland ASVs) (Figure S1). The assembly of the metatranscriptome resulted in 4,217,269 contigs, from which 970,582 contigs (23%) had a taxonomic assignment (MMETSP); these contigs were annotated based on the Pfam database of protein families, which resulted in 17,926 Pfam domains with taxonomic and functional assignment. The Pfam domains represent the conserved functional part of a protein; the metatranscriptome Pfam annotations were used as a proxy to further analyze and compare functional biodiversity based on expressed cellular processes.

The taxonomic assignment of ASVs (rRNA) and contigs (mRNA) resulted in a similar overall distribution of the supergroups alveolates and stramenopiles (Figure S2), but also reached high similarity at a lower taxonomic level (Supplementary ASV Table). This corresponding pattern of taxonomic diversity based on rRNA and mRNA sequence comparison allowed a valid separation of functions into different taxonomic groups based on Pfam annotations. Among the alveolates, 85% ASV and 92% contig reads were assigned to dinoflagellates, and among the stramenopile, 88% ASV and 97% contig reads were assigned to diatoms.

The communities of Icelandic and Swedish coastal water samples were dominated by alveolates, especially dinoflagellates, as revealed by their rRNA and mRNA sequences (Figures S2 and S3). This indicates a seasonal succession from diatoms, which still dominated the microeukaryotic communities in Greenland and Norway, to dinoflagellate-dominated communities. Swedish rRNA samples were dominated by *Lingulodinium* (Gonyaulacales) and *Protoperidinium* (Peridinales) (~48% of assigned reads) and mRNA samples matched mainly *Lingulodinium* (65% of assigned reads). *Protoperidinium* was not detected based on the mRNA, but ~4% of the mRNA reads were assigned to its higher order Peridiniales. Among rRNA samples from Iceland coastal water, *Dinophysis* (Dinophysiales) and *Tripos* (Gonyaulacales) (~39% of assigned reads) were identified and among mRNA samples, *Dinophysis* and *Neoceratium* (Gonyaulacales) were identified (~61% of assigned reads), but *Tripos* was not. The observed discrepancies for *Protoperidinium* and *Tripos* may be explained by the lack of references to these groups in the respective databases.

The communities in Greenlandic and Norwegian water samples were dominated by stramenopiles, as revealed by their rRNA and mRNA sequences. Greenlandic rRNA and mRNA samples mainly consisted of *Detonula*, *Thalassiosira* (both Thalassiosirales) and *Chaetoceros* (Chaetocerales) (63% and 74% of assigned rRNA and mRNA reads, respectively) and the Norwegian rRNA and mRNA samples dominated by *Corethron* (Corethrales) and *Chaetoceros* (Chaetocerales) genera (79% and 64% of assigned reads). Species of the genus *Chaetoceros* were also present in Swedish coastal rRNA water samples (21%) but only accounted for 0.5% of the mRNA signal. This discrepancy could be the result of a PCR bias towards this species group during rRNA amplification or, more likely, may be the result of the insufficient representation of environmentally significant mRNA sequences in the reference database. Overall, the taxonomic assignment deduced from the rRNA and the mRNA was similar. Discrepancies between the taxonomic resolution of the rRNA and mRNA sequences can be expected due to the differences in the completeness of the corresponding reference databases, namely Silva 132 LSU for rRNA [50] and MMETSP for mRNA [57]. For example, some species may be completely absent from one of the databases or only relatives may be represented. Alternatively, different and changing taxonomic affiliations can complicate a clear classification, e.g., the change from *Ceratium* to *Tripos*.

### 3.2. Regional Environmental Conditions and Differences in Taxonomic and Functional Biodiversity

Principle component analysis of environmental parameters (Figure S4) revealed differences between the regions with the most distinguishable environmental conditions between Swedish and Greenland/Norway coastal waters. The clustering of the samples represents the water bodies characterized by the arctic, subarctic or temperate region. Surface water temperatures (Figure S5) were highest in Swedish coastal waters (15–18 °C) and coldest in Greenland (3–7 °C). Mean surface salinity was highest for Iceland and Norway (~34.5) and lowest in Sweden (~24.5) coastal water due to the influence of Baltic Sea water masses. In Greenland coastal water, salinity was generally lower (between 31 and 32) due to arctic freshwater input of melting glaciers and sea-ice but reached levels of 34 at two stations. Generally, low nitrite, nitrate and ammonium concentrations (~0.01–1.16 µM) were measured at all stations and regions; however, overall nitrite concentrations were highest at two Swedish stations (0.3–0.45 µg L^−1^) and total nitrogen concentration was highest in Norway (1.3–4.3 µg L^−1^). Phosphate concentrations tracked silicate concentrations but were lower overall than silicate concentrations (≤0.3 µg L^−1^).

**Figure 2:**
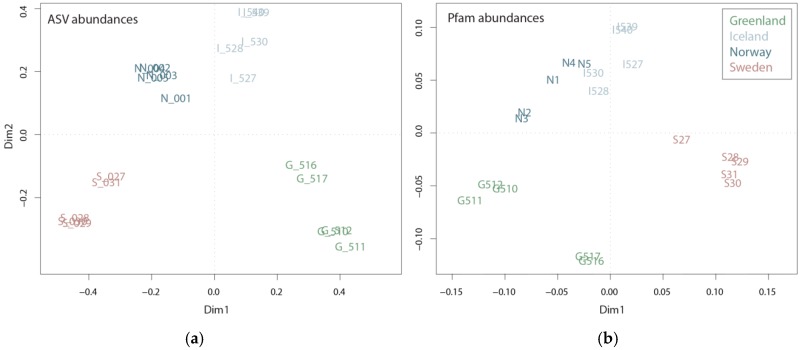
Plots of principal coordinate analysis of amplicon sequence variants (ASV) abundances (**a**) and Pfam domain abundances (**b**) among the sampling sites Greenland, Iceland, Norway and Sweden.

Community composition based on mRNA and rRNA reflected the ecosystem from which the samples originated, namely the arctic coastal waters of Greenland, the subarctic coastal waters of Norway and Iceland and the temperate Swedish coastal waters (Figure 1 and Figure S3). Sampling took place in more or less the same season (27 July–6 September); however, differences in the plankton communities are expected due to successional patterns, i.e., summer plankton succession is further advanced in temperate regions compared to subarctic or arctic regions during the same period. The successional differences between the regions, along with regional differences in prevailing environmental conditions, are well reflected in both taxonomic and functional diversity (Figure 2). The taxonomic diversity shows a clear partitioning between the four regions, with each region located in a separate quadrant of the Principle Coordinate Analysis (PCoA). Accordingly, like the ASV PCoA (Figure 2a), the Pfam domain composition and abundance deduced from mRNA sequences showed partitioning between the four regions (Figure 2b), capturing 37.5% of the variance. However, the samples from the subarctic coastal waters of Norway and Iceland seem to be more similar on a functional level than on a taxonomic level. This is consistent with the results of the water chemistry analysis, which revealed a higher similarity between Iceland and Norway’s coastal water samples (Figures S4 and S5) than between the other two regions. Given the fact that the plankton communities from Norwegian and Icelandic samples are dominated by different taxonomic groups, i.e., diatoms and dinoflagellates, respectively, their functional similarity seems largely influenced by water chemistry and not by taxonomic identity. Because the two groups are dominant among Greenlandic and Norwegian coastal water samples (i.e., diatoms) or Sweden and Icelandic coastal water samples (i.e., dinoflagellates) functional differences should be noticeable since the groups have generally different roles within the ecosystem [13].

### 3.3. Metatranscriptomic Analysis—Functional Biodiversity and Functional Processes Deduced from Metatranscriptomics

KEGG Orthologies (KOs) were assigned to Pfam domains based on the presence of a respective protein domain within a KO gene representative. Although this approach can lead to the loss of information when Pfam domains cannot be matched to KO identifiers, it nonetheless offered the benefit of providing a functional classification of 22% of expressed genes. In sum, from a total of 17,926 Pfam domains, 8135 could be mapped and classified with the KO identifier annotations and subsequently plotted with the FuncTree2 tool for detailed interpretation.

The abundance of all Pfam domains in Swedish, Icelandic, Greenlandic and Norwegian coastal water samples clustered well with the geographic origin of the sample (Figure 2b), e.g., Icelandic samples close to the Norwegian samples. Pfam domain occurrences were plotted as presence/absence data with FuncTree2, which revealed that the metatranscriptomes of all four sampling regions are functionally similar (Figure S6). Hence, based on their functional potential, the communities do not differ greatly, but they differ based on their gene expression profiles (Figure 2b). This implies that it is not the functional diversity stored in a community transcriptome that explains cellular processes associated with certain environmental settings, it is how this functional diversity is manifest, i.e., the genes expressed. The question remains as to what the reasons for this different expression of functional processes may be. For example, it could be the biodiversity within a community or species interactions (e.g., protection against grazing or the secretion of allelochemical molecules). Various environmental parameters influence the transcriptome, as species need to adapt to environmental circumstances.

As environmental parameters often correlate and interact, it is generally difficult to pinpoint one driver of a particular effect. Generally, the abundance of transcripts/sequence reads of a gene reflects the sum of all biotic and abiotic factors acting at the moment of sampling. Also, other stochastic and environmentally independent factors can influence the abundance of a gene in a community metatranscriptome analysis, such as e.g., grazing, parasitism and population size. To gain insight into how certain environmental settings alter functional diversity, we analyzed which parts of the variance of the Pfam domain abundances can be explained by broadly defined biotic and abiotic parameters. In our study, many environmental parameters were correlated (Figure S7); thus, we only associated Pfam domains to a certain environmental parameter if the variance explained by the respective parameter exceeded the sum of variances explained by the other parameters (including the residual unexplained variance).

**Figure 3:**
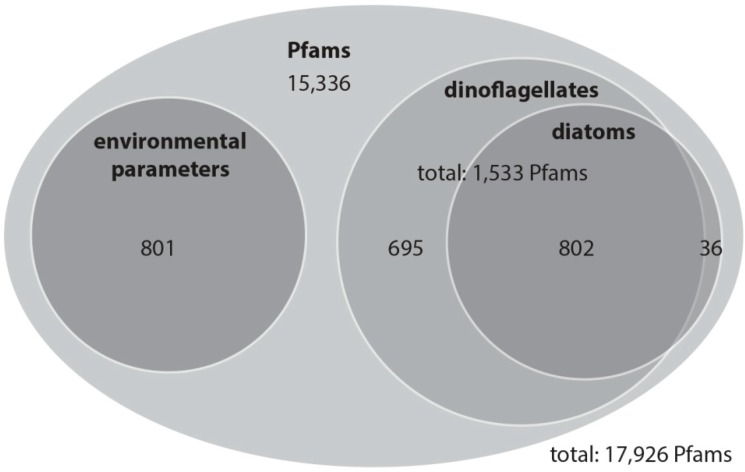
Overview of the Pfam domain distribution as a Venn diagram: numbers represent Pfam domains for which variability is most likely related to changes in environmental parameters (chlorophyll-*a*, phosphate, salinity, silicate or temperature), relative dinoflagellate abundance, relative diatom abundance or both relative dinoflagellate and diatom abundance.

With this rather conservative approach, we identified 801 Pfam domains out of a total of 17,926 domains that can be explained by the environmental variation, and 1533 Pfam domains that explain variation among the community as determined based on the percentage of dinoflagellates and diatoms in the samples. The variation partitioning analysis with dinoflagellates revealed that 1497 Pfam domains are potentially associated with changes in dinoflagellate abundance independent of environmental influences. The same analysis with diatoms revealed that 838 Pfam domains are associated with changes in diatom abundance and are independent of environmental influences. Of these dinoflagellate and diatom Pfam domains, 802 domains are shared (Figure 3). In total 1533 Pfam domains account for dinoflagellates, diatoms or both of these classes.

#### 3.3.1. Functional Response to Environmental Factors

In general, all processes involved in functional pathways play a vital role in species and community interactions. Some functional pathways may be expressed from all species and thus form a general signal, whereas others may be affected by environmental parameters. Here, several Pfam domains (no. = 801) for which the majority of the variance could be attributed to certain environmental factors (temperature, salinity, silicate, phosphate or chlorophyll-*a*) were identified. Most Pfam domains were associated with temperature and salinity; hence, corresponding functions are discussed with an emphasis on these two parameters. The KO terms were assigned to the 801 Pfam domains, but given the situation that not all Pfam domains had a corresponding KO term and some Pfam domains corresponded to multiple KO terms, the environmental parameters silicate, phosphate and chlorophyll-*a* contributed little information (Figure S8) and are not further discussed here (e.g., silicate had only one KO term). A full list of associated Pfam domains can be viewed in the Supplemental Material. The influences of temperature and salinity on functional processes are highlighted and discussed in the following paragraphs.

##### Temperature

The shifts of species distributions towards polar regions due to an increase in temperature at lower latitudes [8] lead to adaptation of local species or the introduction of new species, either of which can have cascading effects in a community. Temperature effects on marine plankton are often described in the context of two fundamental concepts, namely, the metabolic theory of ecology [60,61] and the hypothesis of temperature-dependent physiology [62,63]. Although these two concepts are often studied separately, they are directly linked to each other by the influence of temperature on chemical reactions and metabolic rates, i.e., the sum of all enzyme-driven chemical reactions within cells [64].

Increases in temperature translate into increased rates of cellular and metabolic processes, most of which require ATP. The increased cellular demands result in an increased production of ATP, which is derived from respiration and photosynthesis [61,64,65]. Both processes are affected differently by temperature increases, with respiration possessing a relatively higher activation energy and hence respiration rates should increase more rapidly compared with photosynthesis [61,64,65]. Additionally, temperature is a key determinant of phytoplankton stoichiometry [63,64,66,67], and changes in temperature have been shown to alter the optimal allocation to C, N and P pools in phytoplankton via phenotypic plasticity [63,66,67]. Temperature has been shown to positively correlate with C:P and N:P ratios, but not with C:N. These shifts occur because fewer P-rich ribosomes are required at warmer temperatures due to their increased use efficiency [62,63,67,68]. However, elevated C and N content and a decrease of P have also been attributed to observed stoichiometric changes in warm-acclimated phytoplankton cells [69]. Hence, more than one process potentially causes temperature-dependent shifts in elemental ratios.

**Figure 4:**
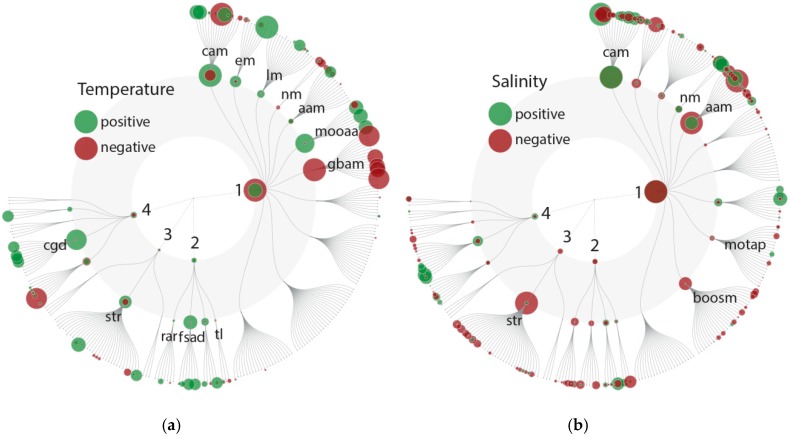
FuncTree 2 plot: positive (green) or negative (red) correlation with Pfam domains depicting variability most likely related to changes in temperature (**a**) and salinity (**b**). The KEGG biologic categories are **metabolism (1)**: carbohydrate metabolism (cam), energy metabolism (em), lipid metabolism (lm), nucleotide metabolism (nm), amino acid metabolism (aam), metabolism of other amino acids (mooaa), gylcan biosynthesis and metabolism (gbam), metabolism of terpenoids and polyketides (motap), biosynthesis of other secondary metabolites (boosm); and **genetic information processing (2)**: replication and repair (rar), folding, sorting and degradation (fsad) and translation (tl); **environmental information processing (3)**: signal transduction (str); and **cellular processes (4)**: cell growth and death (cgd). More details on the KEGG categories can be found in Figure 5. KEGG Orthology (KO) abundance was counted and plotted (size = relative abundance of KOs).

Consistent with a general increase in metabolic activity with temperature, and an associated higher demand for ATP, we found that Pfam domains assignable to the KEGG group carbohydrate metabolism (especially the citrate cycle, glycolysis/gluconeogenesis and pyruvate metabolism) to be positively correlated with temperature (Figure 4a). We also found increased abundances of Pfam domains involved in oxidative phosphorylation. Additionally, we found a positive correlation within the KEGG group metabolism of other amino acids and herein the pathways of glutathione and taurine/hypotaurine metabolism. Glutathione and taurine control the redox gradient across the mitochondrial inner-membrane and act as a pH buffer in the mitochondrial matrix, thereby ensuring performance under high loads of electrons [70]. Further, we found Pfam domains attributable to sulfur metabolism within the KEGG group energy metabolism that positively correlated with temperature. Hence, temperature may not only affect the stoichiometry of C:N:P but also cellular sulfur contents, which can have cascading effects through the marine sulfur cycle.

We also identified patterns that are potentially linked to changes in cellular stoichiometry as proposed by the temperature-dependent physiology hypothesis [62,63]. Consistent with Toseland et al. [67], we found that temperature negatively correlates with the abundance of Pfam domains in the KEGG group translation (ribosome, and biogenesis of ribosomes) and nucleotide metabolism (Figure 4a). Both processes are functionally interlinked, as most substrates from purine and pyrimidine metabolism are used for rRNA synthesis [67,71]. These patterns confirm the molecular explanation for observed declines in cellular P contents with increasing temperature [63,67,68]. Furthermore, parts of our data also include cellular processes that can be attributed to increases in C and N content. These processes include a positive association between temperature and Pfam domain abundances involved in lipid metabolism (one of the major cellular sinks for C allocation) and amino acid metabolism (cyanoamino acids, which represents an N-sink). With increasing temperature and possibly more metabolic activity, it can be further assumed that pathways involved in the growth and turnover of microeukaryotic cells are stimulated. Positive correlations were observed among the KEGG category cellular processes, especially in the group cell growth and death (Figure 4a).

In sum, our results at the genetic level demonstrate higher metabolic activity and respiration with increasing temperatures, as predicted by the metabolic theory of ecology [60,61]. Although we did not measure the C:N:P ratio, our genetic data are consistent with other published results mentioned here, i.e., metabolic processes related to C-, N- and P-allocation change along the temperature gradient (Figure 4a), largely independent of the underlying broad taxonomic composition. This is consistent with what Toseland et al. [67] found at the genetic level and Yvon-Durocher et al. [68] at the community level, namely that there is a highly conserved response of elemental stoichiometry to temperature across multiple spatial, temporal and organizational scales.

In addition to the above-discussed mechanisms, we also observed further indications for higher cellular activity and an associated turnover of proteins due to a positive correlation of Pfam domains involved in the KEGG category genetic information processing, in particular within the groups folding, sorting and degradation and replication and repair. In the category environmental information processing, only a few positive and negative correlations for signal transduction pathways were observed, suggesting that signal transduction may be less influenced by temperature.

##### Salinity

Pfam domains (595) for which most of the variance could be explained by changes in salinity showed rather diverse effects on metabolic processes, with negative and positive associations in various categories (Figure 4b). Salinity influences an organism’s capacity for ion homeostasis. This is associated with salinity having a strong effect on community composition, i.e., indicating that species-specific responses determine fitness variation among community members. We identified both negative and positive correlations between salinity and Pfam domain abundances among the KEGG groups amino acid metabolism, nucleotide metabolism and carbohydrate metabolism (Figure 4b). A more unidirectional negative correlation with salinity was observed for Pfam domains assigned to secondary metabolisms, i.e., biosynthesis of other secondary metabolites (e.g., stilbenoid, diarylheptanoid and gingerol biosynthesis) and metabolism of terpenoids and polyketides (e.g., diterpenoid biosynthesis and biosynthesis of ansamycins). Such effects are well documented in the literature for several plankton groups, e.g., *Prymnesium* [72,73], diatoms [74] and dinoflagellates [75]. Cellular concentrations of many secondary metabolites change at lower salinities, although the direct physiological mechanisms and metabolic linkages remain unknown. Another unidirectional response was observed for the KEGG category environmental information processing at lower salinities, which seems to exert a direct effect on the expression of several signal transduction processes (Figure 4b). This higher expression of signal transduction processes may be a factor underlying the variable expression of metabolic processes in low salinity regimes and further indicates that salinity has an overall strong effect (in comparison to temperature) on the community (also observed among the taxonomic diversity).

#### 3.3.2. Functional Community Response of Diatoms and Dinoflagellates

**Figure 5:**
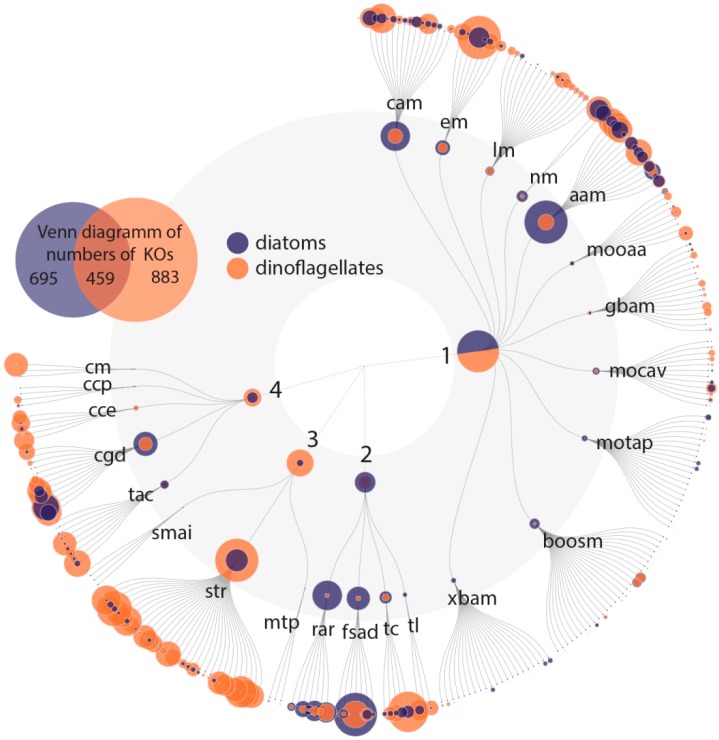
FuncTree 2 Plot: positive correlation with Pfam domains for which variability is most likely related to changes in the abundance of diatoms or dinoflagellates. The KEGG biologic categories are **metabolism (1)**: carbohydrate metabolism (cam), energy metabolism (em), lipid metabolism (lm), nucleotide metabolism (nm), amino acid metabolism (aam), metabolism of other amino acids (mooaa), gylcan biosynthesis and metabolism (gbam), metabolism of cofactors and vitamins (mocav), metabolism of terpenoids and polyketides (motap), biosynthesis of other secondary metabolites (boosm) and xenobiotics biodegradation and metabolism (xbam); **genetic information processing (2):** replication and repair (rar), folding, sorting and degradation (fsad), transcription (tc) and translation (tl); **environmental information processing (3):** signaling molecules and interaction (smai), signal transduction (str) and membrane transport (mrp); and **cellular processes (4):** cell motility (cm), cellular community–prokaryote (ccp)/eukaryotes (cce), cell growth and death (cgd) and transport and catabolism (tac). KO abundance was counted and plotted (size = relative abundance of KOs in each plot). The Venn diagram shows the overlapping KOs between diatoms and dinoflagellates. Some KOs mainly appear in dinoflagellates (e.g., environmental information processing), whereas for genetic information processing some KOs can be found only in diatoms.

Diatoms and dinoflagellates have different life strategies and niches within the marine environment. Diatoms are photosynthetic autotrophs (except for some obligate heterotrophic species of the genus *Nitzschia* [76,77]) with a strong dependency on inorganic nutrients, whereas dinoflagellates can have different trophic niches and can be more variable in their trophic roles. In this study, the different cellular community functions of diatoms and dinoflagellates were assessed by using the abundance of Pfam domains as a proxy. The KO terms were assigned to Pfam domains for which most of the variance could be attributed to changes in the relative abundance of diatoms and dinoflagellates and were plotted with FuncTree2 (Figure 5). As a result of the strong negative correlation of dinoflagellate and diatom abundance in our data set (Figure S9), we applied variance partitioning to each separately and combined the result afterward (see Material and Methods). This means, that for most of the data, most of the variance could either be explained by an increase in dinoflagellate abundance and a decrease in diatom abundance or vice versa (Figure S9). Any described positive association with diatom abundance hereafter refers to processes that increase with diatom abundance and decrease with dinoflagellate abundance. Therefore, only the positive correlation of the Pfam domain variability is plotted (Figure 5). Almost half of the Pfam domains, for which most of the variance was explained by shifting taxa abundances, were only associated with changes in dinoflagellate abundance. Hence, some Pfam domain abundances fit well with the dinoflagellate abundance alone and were less influenced by the other tested parameters.

In general, cellular functions and metabolic pathways associated with diatoms and dinoflagellates abundance relate to the same processes, but they are differentially expressed (Figure 5). Both groups are phylogenetically and evolutionary distinct [78], which has resulted (among other things) in differences in gene structure and regulation. The distinct genetic diversity between the two groups together with interspecific interactions may additionally affect the gene regulation as response to e.g., protection against grazing or the secretion of allelochemical active molecules (e.g., toxins). Differentially evolved regulations of cellular and physiological functions may promote different lifestyles. Similar environmental signals lead to taxa-specific differential regulation of the same pathways, hence creating different cellular responses and trade-offs.

##### The Metabolic Associations with Diatoms and Dinoflagellates

In the biologic KEGG category metabolism, KOs of dinoflagellates and diatoms were positively associated, especially with pathways of carbohydrate metabolism (e.g., amino sugar and nucleotide sugar metabolism, ascorbate and aldarate metabolism, the citrate cycle and pentose phosphate pathway), energy metabolism (e.g., carbon fixation in photosynthetic organisms, methane metabolism, photosynthesis), nucleotide metabolism (purine and pyrimidine metabolism) and amino acid metabolism (e.g., alanine, aspartate and glutamate metabolism, arginine and proline metabolism, cysteine and methionine metabolism, glycine, serine and threonine metabolism and valine leucine and isoleucine degradation) (Figure 5). Upregulated genes involved in carbohydrate metabolism are associated with the dinoflagellate *Prorocentrum donghaiense* and with diatoms [25]. Furthermore, positive correlations were observed for the dinoflagellate abundance with the catabolic process beta-oxidation of lipid metabolism. Diatom abundance correlated positively with fatty acid synthesis (KEGG pathway fatty acid biosynthesis, elongation), which is an anabolic process. This indicates that dinoflagellates preferably use the catabolic and diatoms, as autotrophs, the anabolic part of fatty acid metabolism. Additionally, diatoms can accumulate lipids, which are more lightweight than other chemical constituents and thus regulate their buoyancy [79]. This extra buoyancy (compared with other organisms) may counteract the effect of their relatively heavy silica cell walls.

Among the metabolic processes, diatom abundance correlated positively with lysine metabolism (in KEGG group amino acid metabolism), especially lysine degradation, with fatty acid synthesis (fatty acid biosynthesis, elongation) and with the C5 isoprenoid biosynthesis, mevalonate (MVA) pathway. The degradation of lysine delivers Acetyl-CoA, a universal metabolic precursor for several pathways, including fatty acid biosynthesis and *C5* isoprenoid biosynthesis, MVA pathway. Lysine was shown to be actively taken up from media by the model diatom species, *Phaeodactylum tricornutum* to complement N and C demand [80,81]. The ability to take up lysine may not be present in all diatom species and the contribution and availability of lysine to overall C and N demand remains to be determined. However, the positive association of diatoms with lysine degradation observed here indicates the physiological importance of this metabolic pathway for diatoms in their natural environment.

The positive association of diatoms with fatty acid synthesis and with the C5 isoprenoid biosynthesis, the MVA pathway can have several, non-mutually exclusive reasons. Both, the fatty acid synthesis and the C5 isoprenoid biosynthesis, MVA pathway are the starting points for a rich suite of metabolites, including carotenoids, sterols, isoprenoids and hydrocarbons. The presence of these compounds in diatoms has put them on the research agenda as a source of natural products and as a proxy for climate reconstruction [79,82]. Our result, therefore, stresses the ecological relevance of these compounds in diatoms and hence the importance of diatoms as a producer of these compounds for other trophic levels. Carotenoids, for example, are known to be produced in large quantities in diatoms [83] and serve several functions at higher trophic levels, e.g., as antioxidants due to their effects on the immune system and involvement in vision or body coloration [84]. Diatoms are further known to contribute a major proportion of marine volatile isoprene, which can have regional effects on oceanic climate [85,86]. The positive association of diatoms with the metabolic pathway genes of these compounds in our study is not surprising but has not yet been described before using metatranscriptomics data from field samples. Our results show that genes of metabolic pathways, for which diatoms have already been described as dominant producers, can be traced back to genetic activity patterns in field samples. This illustrates the ecological relevance of these metabolic pathways and compounds, as they are linked to the success of diatoms. Accordingly, these pathways are relevant for other trophic levels and ecosystem functions via diatoms.

For dinoflagellates, the positive association with nitrogen metabolism (within the KEGG category energy metabolism) was particularly notable among the metabolic processes. This group contained genes coding for nitrate/nitrite transporters, nitrate reductase, ammonium assimilating enzymes (glutamate dehydrogenase, glutamate synthase) and genes coding for cyanate lyases. These results correspond well with the results of a metatranscriptome study by Zhuang et al. [87], who identified the same nitrogen acquisition genes associated with a dinoflagellate bloom (*Alexandrium fundyense,* now *A. catenella* [88]). Particularly striking is the congruent identification of the positive association of the cyanate lyase gene with the abundance of dinoflagellates, which provides further evidence that the use of cyanate as an organic N-source is an ecologically important trait in dinoflagellates [87]. Dinoflagellates mainly dominate plankton communities at times when macronutrients are limiting. A positive association of dinoflagellates with N-acquisition genes was linked to growth promotion, with the exploitation of various N-sources (DIN, DON) likely conferring a competitive advantage [87].

##### Other Functional Associations with Diatoms and Dinoflagellates

In the biologic KEGG category cellular processes, among cell growth and death (i.e., cell cycle, meiosis), positive correlations were shared with dinoflagellates and diatoms, but most other Pfam domains in this category were positively correlated with dinoflagellates. As expected, since planktonic diatoms are not mobile, no KO terms of cell motility processes correlated positively with this group. The overall positive correlations of dinoflagellates with several cellular processes are most likely related to the broad functional diversity of this group. Transport and catabolism processes were positively correlated with dinoflagellates, especially to autophagy and endocytosis. These two processes are closely linked to vesicular transport. For the biologic KEGG category genetic information processing (folding, sorting and degradation), dinoflagellates highly correlated to SNARE interactions in vesicular transport (SNARE: SNP-Receptor, Soluble N-ethylmaleimide sensitive fusion attachment protein receptor). Pathways related to the uptake and secretion of substances (intracellular trafficking, secretion and vesicular transport; KOG-Category ‘U’) were also identified in a metatranscriptomics study by Wohlrab et al. [23] as particularly important for the persistence of dinoflagellate communities. Also, important are processes associated with the excretion or uptake of substances that can have profound effects on community members and biogeochemical processes (e.g., the secretion of allelochemically active molecules).

The biologic KEGG category genetic information processing was split into two groups: a group of pathways with more KOs positively correlating with the variation in diatoms compared to dinoflagellates and the converse for the other group. Diatom abundance correlated in pathways of replication and repair, as well as folding, sorting and degradation, probably reflecting their characteristic behavior as r-strategists with high growth rates [89]. Dinoflagellate abundance correlated with pathways of transcription and (to a lesser degree) translation. Also, dinoflagellates were positively associated with the biologic category of environmental information processing and especially signal transduction pathways. These positive associations reflect the transcriptomic characteristics of dinoflagellates, which possess genes coding for protein kinases, EF-hand domain containing proteins (both involved in signal transduction) and RNA recognition factors (involved in transcription) [90].

Compared to other biologic categories and pathways, the differences between the dinoflagellates and diatoms were most evident within the environmental information processing category. The broader diversity of lifestyles of dinoflagellates compared with diatoms strongly reflects biotic interactions [91,92]. Signal transduction processes are thereby a prerequisite for the interaction between eukaryotic microalgae and other components of the marine food web, thereby affecting energy flow, food web dynamics, and community structure [93]. Dinoflagellates, and in particular toxigenic species, can use different (species-specific) forms of biologically active secondary metabolites, which, once excreted, interact with cell membranes and affect signal transduction at receptor sites of target organisms [93]. Species interactions, therefore, have played a central role in the evolution of dinoflagellates and have shaped their gene content, as demonstrated by the results of metatranscriptomics analysis in this study and several previously reported several transcriptomic studies.

## 4. Conclusions

**Figure 6:**
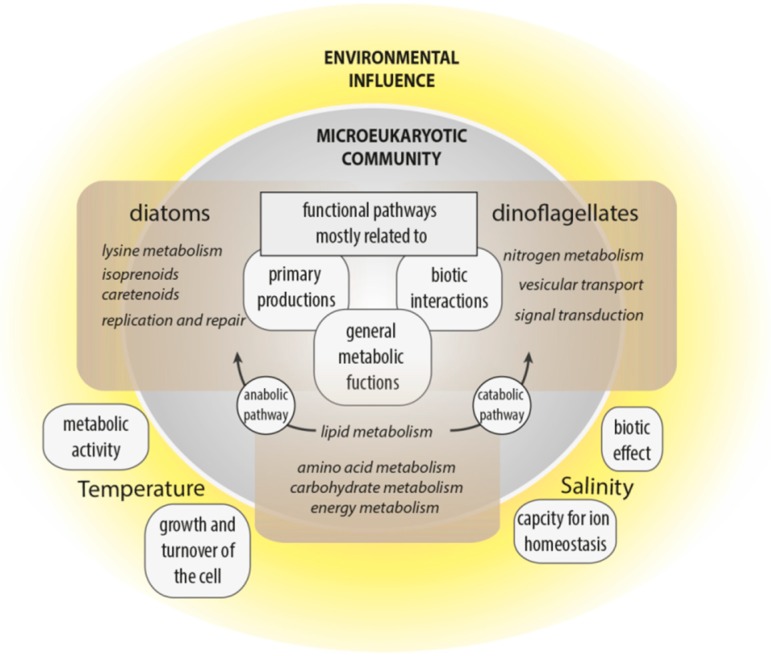
Summary of the main influences of temperature and salinity on the microeukaryotic community, and main functional pathways correlated with diatom or dinoflagellate abundance.

This study aimed to improve understanding of the interplay of community composition, functional traits and environmental parameters to strengthen the hypothesis that marine communities and ultimately higher trophic levels are affected by the environmental conditions associated with climate change. Variance analyses of Pfam domains deduced from the metatranscriptomes elucidated functional cellular and metabolic processes affected by changing environmental conditions or community structure, reflecting aspects of the ecological niches of dinoflagellates and diatoms (Figure 6).

Temperature and salinity had the most influence on the functional biodiversity of microeukaryotic communities in the arctic and subarctic/temperate waters. However, the clear partitioning of taxonomic diversity between the four regions (especially Norwegian and Icelandic samples), is not consistent with their functional similarity, which seems largely to be influenced by water chemistry and not by taxonomic identity. Thus, despite the overall geographic similarity of functional responses, the identified abiotic (i.e., temperature and salinity) and biotic (i.e., taxonomic group, namely diatoms and dinoflagellates) factors influenced functional responses. Hence, cellular processes associated with certain environmental settings originate less from the functional diversity stored in a community’s transcriptome than how this functional diversity is expressed.

Functional processes were significantly influenced by temperature and salinity. Temperature is a primary driver of metabolic activities and growth, while salinity influences an organisms’ capacity for ion homeostasis. The general increase in metabolic activity with temperature and an associated higher demand for ATP was associated with metabolic processes related to C-, N- and P-allocation shifts along the temperature gradient, largely independent of the underlying diverse taxonomic composition (i.e., positive correlations with carbohydrate metabolism, especially the citrate cycle, glycolysis/gluconeogenesis and pyruvate metabolism). Further, the tendency for higher growth was evidenced by a positive correlation between temperature and the category cellular processes, especially in the group of cell growth and death. Along the salinity gradient, differences in the expression of most metabolic functions were identified: secondary metabolism correlated positively, and signal transduction correlated negatively with increased salinity. Hence, salinity had an overall strong effect (in comparison with temperature) on the community, as well as on taxonomic diversity.

Cellular functions and metabolic pathways of diatoms and dinoflagellates were associated with the same genetically based structure, but genes were differentially expressed. Differentially evolved regulations of cellular and physiological functions may promote different life-history strategies. Similar environmental signals possibly lead to taxa-specific differential regulations of the same pathways, hence creating different cellular responses and trade-offs. For dinoflagellates, the importance of nitrogen metabolism, vesicular transport, and signal transduction highlights the role of biotic interactions in the evolution of dinoflagellates. Further, this study shows that the expression of various N-acquisition genes in dinoflagellates is correlated with their abundance; hence, the exploitation of various N-sources likely confers a competitive advantage on this group. Diatoms were especially associated with metabolites, like isoprenoids and carotenoids, which highlights their critical autotrophic role. Diatoms were also linked with lysine degradation, indicating the physiological importance of this metabolic pathway for diatoms in their natural environment.

Both, the influence of the environment on the microeukaryotic community, as well as functional processes influenced by the diversity, impact the overall marine diversity and higher trophic levels; however, the amount of impact has to be examined. The information gathered here can support ecological questions concerning the marine ecosystem state and metabolic interactions in the marine environment.

## Supplementary Materials

The following are available online at https://www.mdpi.com/2076-2607/8/4/567/s1, Figure S1: Venn diagram of ASVs among the microeukaryotes in the four coastal sampling regions, Figure S2: Bar chart of the relative rRNA and mRNA diversity of the microeukaryotes, Figure S3: Distribution of taxonomic groups in the four coastal sampling regions, Figure S4: Principle Component analysis (PCA) of Euclidean distances of environmental parameters, Figure S5: Distribution of environmental parameters of the sampling regions, Figure S6: FuncTree2 Plots of all Pfam domains corresponding to KEGG Orthologies (KO) terms for each region, Figure S7: Heat map of correlation between environmental parameters and relative abundance of dinoflagellates and diatoms, Figure S8: FuncTree 2 Plot of correlations with Pfam domains and what the variability is most likely related to, including chlorophyll-a, phosphate, and silicate, Figure S9: The negative correlation between dinoflagellates and diatom Pfam abundancetitle, Table S1: Overview of 454-pyrosequencing reads and number of ASVs, Table S2: Details to supplementary Figure S5: Environmental parameters concentrations.

## References

[1] Baas-Becking L.G.M. (1934). Geobiologie of Inleiding tot de Milieukunde.

[2] Longhurst A.R. (2007). Ecological Geography of the Sea.

[3] Lechtenfeld O.J., Kattner G., Flerus R., McCallister S.L., Schmitt-Kopplin P., Koch B.P. (2014). Molecular transformation and degradation of refractory dissolved organic matter in the Atlantic and Southern Ocean. Geochim. Cosmochim. Acta.

[4] Barton A.D., Dutkiewicz S., Flierl G., Bragg J., Follows M.J. (2010). Patterns of diversity in marine phytoplankton. Science.

[5] De Vargas C., Audic S., Henry N., Decelle J., Mahé F., Logares R., Lara E., Berney C., Bescot N., Probert I. (2015). Eukaryotic plankton diversity in the sunlit ocean. Science.

[6] Hanson C.A., Fuhrman J.A., Horner-Devine M.C., Martiny J.B.H. (2012). Beyond biogeographic patterns: Processes shaping the microbial landscape. Nat. Rev. Microbiol..

[7] Martiny J.B.H., Bohannan B.J.M., Brown J.H., Colwell R.K., Fuhrman J.A., Green J.L., Horner-Devine M.C., Kane M., Krumins J.A., Kuske C.R. (2006). Microbial biogeography: Putting microorganisms on the map. Nat. Rev. Microbiol..

[8] Thomas M.K., Kremer C.T., Klausmeier C.A., Litchman E. (2012). A global pattern of thermal adaptation in marine phytoplankton. Science.

[9] Hillebrand H., Blasius B., Borer E.T., Chase J.M., Downing J.A., Eriksson B.K., Filstrup C.T., Harpole W.S., Hodapp D., Larsen S. (2018). Biodiversity change is uncoupled from species richness trends: Consequences for conservation and monitoring. J. Appl. Ecol..

[10] Hillebrand H., Langenheder S., Lebret K., Lindström E., Östman Ö., Striebel M. (2018). Decomposing multiple dimensions of stability in global change experiments. Ecol. Lett..

[11] Litchman E., de Tezanos Pinto P., Klausmeier C.A., Thomas M.K., Yoshiyama K. (2010). Linking traits to species diversity and community structure in phytoplankton. Hydrobiologia.

[12] Petchey O.L., Gaston K.J. (2006). Functional diversity: Back to basics and looking forward. Ecol. Lett..

[13] Glibert P.M. (2016). Margalef revisited: A new phytoplankton mandala incorporating twelve dimensions, including nutritional physiology. Harmful Algae.

[14] Litchman E., Klausmeier C.A., Schofield O.M., Falkowski P.G. (2007). The role of functional traits and trade-offs in structuring phytoplankton communities: Scaling from cellular to ecosystem level. Ecol. Lett..

[15] Litchman E., Klausmeier C.A. (2008). Trait-based community ecology of phytoplankton. Annu. Rev. Ecol. Evol. Syst..

[16] Naeem S., Duffy J.E., Zavaleta E. (2012). The Functions of Biological Diversity in an Age of Extinction. Science.

[17] Mouillot D., Graham N.A.J., Villéger S., Mason N.W.H., Bellwood D.R. (2013). A functional approach reveals community responses to disturbances. Trends Ecol. Evol..

[18] Collins S., Rost B., Rynearson T.A. (2014). Evolutionary potential of marine phytoplankton under ocean acidification. Evol. Appl..

[19] Mouillot D., Villéger S., Scherer-Lorenzen M., Mason N.W.H. (2011). Functional structure of biological communities predicts ecosystem multifunctionality. PLoS ONE.

[20] Mouillot D., Spatharis S., Reizopoulou S., Laugier T., Sabetta L., Basset A., Chi T. (2006). Do Alternatives to taxonomic-based approaches to assess changes in transitional water communities. Aquat. Conserv. Mar. Freshw. Ecosyst..

[21] Beaugrand G. (2005). Monitoring pelagic ecosystems using plankton indicators. ICES J. Mar. Sci..

[22] Pearson G.A., Lago-Leston A., Cánovas F., Cox C.J., Verret F., Lasternas S., Duarte C.M., Agusti S., Serrão E.A. (2015). Metatranscriptomes reveal functional variation in diatom communities from the Antarctic Peninsula. ISME J..

[23] Wohlrab S., Falcke J.M., Lin S., Zhang H., Neuhaus S., Elferink S., Voss D., Zielinski O., John U. (2018). Metatranscriptome Profiling Indicates Size-Dependent Differentiation in Plastic and Conserved Community Traits and Functional Diversification in Dinoflagellate Communities. Front. Mar. Sci..

[24] Zhang F., He J., Lin L., Jin H. (2015). Dominance of picophytoplankton in the newly open surface water of the central Arctic Ocean. Polar Biol..

[25] Zhang Y., Lin X., Shi X., Lin L., Luo H., Li L., Lin S. (2019). Metatranscriptomic Signatures Associated With Phytoplankton Regime Shift From Diatom Dominance to a Dinoflagellate Bloom. Front. Microbiol..

[26] Alexander H., Rouco M., Haley S.T., Wilson S.T., Karl D.M., Dyhrman S.T. (2015). Functional group-specific traits drive phytoplankton dynamics in the oligotrophic ocean. Proc. Natl. Acad. Sci. USA.

[27] Elferink S., Neuhaus S., Wohlrab S., Toebe K., Voß D., Gottschling M., Lundholm N., Krock B., Koch B.P., Zielinski O. (2017). Molecular diversity patterns among various phytoplankton size-fractions in West Greenland in late summer. Deep. Res. Part I Oceanogr. Res. Pap..

[28] Gwenn G.M., Limón M.D.H., Haley S.T., Juhl A.R., Dyhrman S.T. (2017). Diverse CO2-induced responses in physiology and gene expression among eukaryotic phytoplankton. Front. Microbiol..

[29] von Dassow P., John U., Ogata H., Probert I., Bendif E.M., Kegel J.U., Audic S., Wincker P., Da Silva C., Claverie J.-M. (2015). Life-cycle modification in open oceans accounts for genome variability in a cosmopolitan phytoplankton. ISME J..

[30] Wohlrab S., Iversen M.H., John U. (2010). A molecular and co-evolutionary context for grazer induced toxin production in Alexandrium tamarense. PLoS ONE.

[31] John U., Tillmann U., Hülskötter J., Alpermann T.J., Wohlrab S., Van de Waal D.B. (2015). Intraspecific facilitation by allelochemical mediated grazing protection within a toxigenic dinoflagellate population. Proc. R. Soc. B Biol. Sci..

[32] Guidi L., Chaffron S., Bittner L., Eveillard D., Larhlimi A., Roux S., Darzi Y., Audic S., Berline L., Brum J.R. (2016). Plankton networks driving carbon export in the oligotrophic ocean. Nature.

[33] Carradec Q., Pelletier E., Da Silva C., Alberti A., Seeleuthner Y., Blanc-Mathieu R., Lima-Mendez G., Rocha F., Tirichine L., Labadie K. (2018). A global ocean atlas of eukaryotic genes. Nat. Commun..

[34] Alexander H., Jenkins B.D., Rynearson T.A., Dyhrman S.T. (2015). Metatranscriptome analyses indicate resource partitioning between diatoms in the field. Proc. Natl. Acad. Sci. USA.

[35] Marchetti A., Schruth D.M., Durkin C.A., Parker M.S., Kodner R.B., Berthiaume C.T., Morales R., Allen A.E., Armbrust E.V. (2012). Comparative metatranscriptomics identifies molecular bases for the physiological responses of phytoplankton to varying iron availability. Proc. Natl. Acad. Sci. USA.

[36] Dupont C.L., McCrow J.P., Valas R., Moustafa A., Walworth N., Goodenough U., Roth R., Hogle S.L., Bai J., Johnson Z.I. (2015). Genomes and gene expression across light and productivity gradients in eastern subtropical Pacific microbial communities. ISME J..

[37] Ottesen E.A., Young C.R., Gifford S.M., Eppley J.M., Marin R., Schuster S.C., Scholin C.A., DeLong E.F. (2014). Multispecies diel transcriptional oscillations in open ocean heterotrophic bacterial assemblages. Science.

[38] Cembella A.D., Zielinski O., Anderson D.M., Graeve M., Henkel R., John U., Kattner G., Koch B.P., Krock B., Meier D. (2016). ARCHEMHAB: Interactions and feedback mechanisms between hydrography, geochemical signatures and microbial ecology, with a focus on HAB species diversity, biogeography and dynamics—Cruise No. MSM 21/3—July 25—August 10, 2012—Nuuk (Greenland)—Reyk.

[39] Armstrong F.A.J., Stearns C.R., Strickland J.D.H. (1967). The measurement of upwelling and subsequent biological process by means of the Technicon Autoanalyzer® and associated equipment. Deep Sea Res. Oceanogr. Abstr..

[40] Koroleff F. (1969). Direct Determination of Ammonia in Natural Waters as Indophenol Blue.

[41] Grasshoff K., Ehrhardt M., Kremling K., Grasshoff K., Ehrhardt M., Kremling K. (1983). Methods of Seawater Analysis.

[42] Eberlein K., Kattner G. (1987). Automatic method for the determination of ortho-phosphate and total dissolved phosphorus in the marine environment. Fresenius’ Zeitschrift für Anal. Chemie.

[43] Zielinski O., Voß D., Meier D., Henkel R., Holinde L., Garaba S.P., Cembella A. (2013). Chlorophyll a during Maria S. Merian cruise MSM21/3 (ARCHEMHAB). Allan Cembella.

[44] Reeder J., Knight R. (2009). The “rare biosphere”: A reality check. Nat. Methods.

[45] Amir A., McDonald D., Navas-Molina J.A., Kopylova E., Morton J.T., Zech Xu Z., Kightley E.P., Thompson L.R., Hyde E.R., Gonzalez A. (2017). Deblur Rapidly Resolves Single-Nucleotide Community Sequence Patterns. mSystems.

[46] Callahan B.J., McMurdie P.J., Holmes S.P. (2017). Exact sequence variants should replace operational taxonomic units in marker-gene data analysis. ISME J..

[47] Callahan B.J., McMurdie P.J., Rosen M.J., Han A.W., Johnson A.J.A., Holmes S.P. (2016). DADA2: High-resolution sample inference from Illumina amplicon data. Nat. Methods.

[48] Martin M. (2011). Cutadapt removes adapter sequences from high-throughput sequencing reads. EMBnet. J..

[49] R CoreTeam (2016). R: A Language and Environment for Statistical Computing.

[50] Glöckner F.O., Yilmaz P., Quast C., Gerken J., Beccati A., Ciuprina A., Bruns G., Yarza P., Peplies J., Westram R. (2017). 25 years of serving the community with ribosomal RNA gene reference databases and tools. J. Biotechnol..

[51] Vergin K.L., Beszteri B., Monier A., Thrash J.C., Temperton B., Treusch A.H., Kilpert F., Worden A.Z., Giovannoni S.J. (2013). High-resolution SAR11 ecotype dynamics at the Bermuda Atlantic Time-series Study site by phylogenetic placement of pyrosequences. ISME J..

[52] Oksanen J., Blanchet F.G., Kindt R., Legendre P., Minchin P.R., O’Hara R.B., Simpson G.L., Solymos P., Stevens M.H.H., Wagner H. (2013). Package ‘vegan’. Community Ecol. Package Version.

[53] Wickham H. (2011). The split-apply-combine strategy for data analyisis. J. Stat. Softw..

[54] McMurdie P.J., Holmes S. (2014). Waste not, want not: Why rarefying microbiome data is inadmissible. PLoS Comput. Biol..

[55] Legendre P., Gallagher E.D. (2001). Ecologically meaningful transformations for ordination of species data. Oecologia.

[56] Rao C.R. (1995). A review of canonical coordinates and an alternative to correspondence analysis using hellinger distance. Questiió Quad. d’Estadística Sist. Inform. i Investig. Oper..

[57] Keeling P.J., Burki F., Wilcox H.M., Allam B., Allen E.E., Amaral-Zettler L.A., Armbrust E.V., Archibald J.M., Bharti A.K., Bell C.J. (2014). The Marine Microbial Eukaryote Transcriptome Sequencing Project (MMETSP): Illuminating the Functional Diversity of Eukaryotic Life in the Oceans through Transcriptome Sequencing. PLoS Biol..

[58] Ritchie M.E., Phipson B., Wu D., Hu Y., Law C.W., Shi W., Smyth G.K. (2015). Limma powers differential expression analyses for RNA-sequencing and microarray studies. Nucleic Acids Res..

[59] Darzi Y., Yamate Y., Yamada T. (2019). FuncTree2: An interactive radial tree for functional hierarchies and omics data visualization. Bioinformatics.

[60] Brown J.H., Gillooly J.F., Allen A.P., Savage V.M., West G.B. (2004). Toward a metabolic theory of ecology. Ecology.

[61] Allen A.P., Gillooly J.F., Brown J.H. (2005). Linking the global carbon cycle to individual metabolism. Funct. Ecol..

[62] Woods H.A., Makino W., Cotner J.B., Hobbie S.E., Harrison J.F., Acharya K., Elser J.J. (2003). Temperature and the chemical composition of poikilothermic organisms. Funct. Ecol..

[63] Yvon-Durocher G., Dossena M., Trimmer M., Woodward G., Allen A.P. (2015). Temperature and the biogeography of algal stoichiometry. Glob. Ecol. Biogeogr..

[64] Boscolo-Galazzo F., Crichton K.A., Barker S., Pearson P.N. (2018). Temperature dependency of metabolic rates in the upper ocean: A positive feedback to global climate change?. Glob. Planet. Change.

[65] Chen B., Landry M.R., Huang B., Liu H. (2012). Does warming enhance the effect of microzooplankton grazing on marine phytoplankton in the ocean?. Limnol. Oceanogr..

[66] Daines S.J., Clark J.R., Lenton T.M. (2014). Multiple environmental controls on phytoplankton growth strategies determine adaptive responses of the N:P ratio. Ecol. Lett..

[67] Toseland A., Daines S.J., Clark J.R., Kirkham A., Strauss J., Uhlig C., Lenton T.M., Valentin K., Pearson G.A., Moulton V. (2013). The impact of temperature on marine phytoplankton resource allocation and metabolism. Nat. Clim. Chang..

[68] Yvon-Durocher G., Schaum C.E., Trimmer M. (2017). The temperature dependence of phytoplankton stoichiometry: Investigating the roles of species sorting and local adaptation. Front. Microbiol..

[69] Martiny A.C., Ma L., Mouginot C., Chandler J.W., Zinser E.R. (2016). Interactions between thermal acclimation, growth rate, and phylogeny influence prochlorococcus elemental stoichiometry. PLoS ONE.

[70] Hansen S.H., Grunnet N. (2013). Taurine, Glutathione and Bioenergetics. Taurine 8.

[71] Warner J.R. (1999). The economics of ribosome biosynthesis in yeast. Trends Biochem. Sci..

[72] Freitag M., Beszteri S., Vogel H., John U. (2011). Effects of physiological shock treatments on toxicity andpolyketide synthase gene expression in *prymnesium parvum* (prymnesiophyceae). Eur. J. Phycol..

[73] Granéli E., Salomon P.S. (2010). Factors influencing allelopathy and toxicity in prymnesium parvum. J. Am. Water Resour. Assoc..

[74] Rowland S.J., Allard W.G., Belt S.T., Massé G., Robert J.M., Blackburn S., Frampton D., Revill A.T., Volkman J.K. (2001). Factors influencing the distributions of polyunsaturated terpenoids in the diatom, Rhizosolenia setigera. Phytochemistry.

[75] Peter C., Krock B., Cembella A. (2018). Effects of salinity variation on growth and yessotoxin composition in the marine dinoflagellate Lingulodinium polyedra from a Skagerrak fjord system (western Sweden). Harmful Algae.

[76] Li C.W., Volcani B.E. (1987). Four new apochlorotic diatoms. Br. Phycol. J..

[77] Armstrong E., Rogerson A., Leftley J.W. (2000). Utilisation of seaweed carbon by three surface-associated heterotrophic protists, *Stereomyxa ramosa*, *Nitzschia alba* and *Labyrinthula* sp.. Aquat. Microb. Ecol..

[78] Keeling P.J., Burki F. (2019). Progress towards the Tree of Eukaryotes. Curr. Biol..

[79] Stonik V.S., Stonik I. (2015). Low-molecular-weight metabolites from diatoms: Structures, biological roles and biosynthesis. Mar. Drugs.

[80] Flynn K.J., Syrett P.J. (1985). Development of the ability to take up L-lysine by the diatom *Phaeodactylum tricornutum*. Mar. Biol..

[81] Flynn K.J., Syrett P.J. (1986). Utilization of L-lysine and L-arginine by the diatom Phaeodactylum tricornutum. Mar. Biol..

[82] Conte M., Lupette J., Seddiki K., Meï C., Dolch L.J., Gros V., Barette C., Rébeillé F., Jouhet J., Maréchal E. (2018). Screening for biologically annotated drugs that trigger triacylglycerol accumulation in the diatom Phaeodactylum. Plant Physiol..

[83] Levering J., Broddrick J., Dupont C.L., Peers G., Beeri K., Mayers J., Gallina A.A., Allen A.E., Palsson B.O., Zengler K. (2016). Genome-scale model reveals metabolic basis of biomass partitioning in a model diatom. PLoS ONE.

[84] de Carvalho C.C.C.R., Caramujo M.J. (2017). Carotenoids in aquatic ecosystems and aquaculture: A colorful business with implications for human health. Front. Mar. Sci..

[85] Srikanta Dani K.G., Silva Benavides A.M., Michelozzi M., Peluso G., Torzillo G., Loreto F. (2017). Relationship between isoprene emission and photosynthesis in diatoms, and its implications for global marine isoprene estimates. Mar. Chem..

[86] Vavitsas K., Fabris M., Vickers C.E. (2018). Terpenoid metabolic engineering in photosynthetic microorganisms. Genes.

[87] Zhuang Y., Zhang H., Hannick L., Lin S. (2015). Metatranscriptome profiling reveals versatile n-nutrient utilization, co2 limitation, oxidative stress, and active toxin production in an Alexandrium fundyense bloom. Harmful Algae.

[88] John U., Litaker R.W., Montresor M., Murray S., Brosnahan M.L., Anderson D.M. (2014). Formal revision of the alexandrium tamarense species complex (dinophyceae) taxonomy: The introduction of five species with emphasis on molecular-based (rDNA) classification. Protist.

[89] Malviya S., Scalco E., Audic S., Vincent F., Veluchamy A., Bittner L., Poulain J., Wincker P., Iudicone D., de Vargas C. (2016). Insights into global diatom distribution and diversity in the world’s ocean. Proc. Natl. Acad. Sci. USA.

[90] Stephens T.G., Ragan M.A., Bhattacharya D., Chan C.X. (2018). Core genes in diverse dinoflagellate lineages include a wealth of conserved dark genes with unknown functions. Sci. Rep..

[91] Hackett J.D., Anderson D.M., Erdner D.L., Bhattacharya D. (2004). Dinoflagellates: A remarkable evolutionary experiment. Am. J. Bot..

[92] Taylor F.J.R. (2004). Extraordinary Dinoflagellates: Past and Present. Proceedings of International Symposium on “Dawn of a New Natural History-Integration of Geoscience and Biodiversity Studies”.

[93] Cembella A.D. (2003). Chemical ecology of eukaryotic microalgae in marine ecosystems. Phycologia.

