# Supplementary material for: Functional Genomics Differentiate Inherent and Environmentally Influenced Traits in Dinoflagellate and Diatom Communities
Source: Microorganisms. 2020 Apr 15;8(4):567. doi: 10.3390/microorganisms8040567 (PMC7232425; doi:10.3390/microorganisms8040567)
Supplement: Supplementary file 1 [file microorganisms-08-00567-s001.zip › Supplement Figures_2020.04.pdf]

Supplementary figures to

“Functional genomics differentiate inherent and environmentally influenced traits in dinoflagellate and diatom communities”

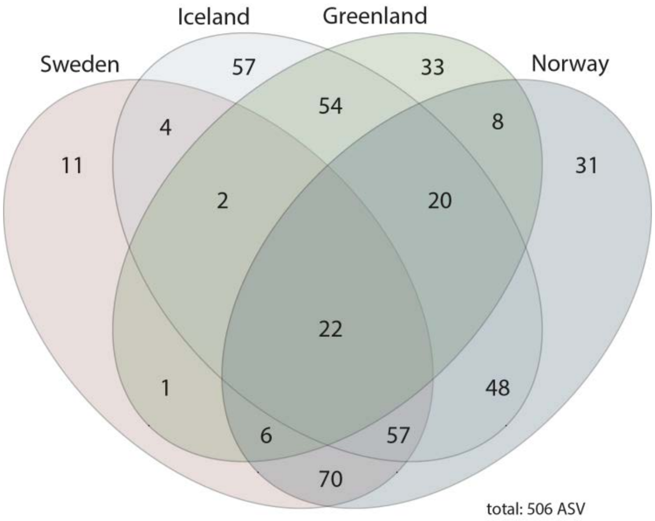

Figure S1: Venn diagram of all 506 ASVs among the microeukaryotes in the four coastal sampling regions: Sweden, Iceland, Greenland, and Norway.

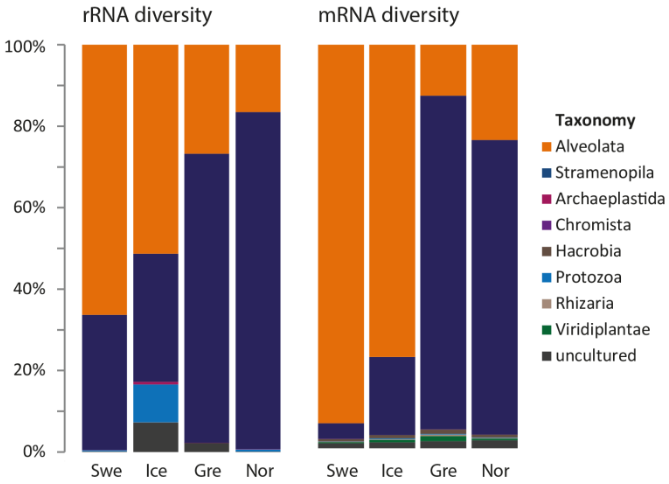

Figure S2: Bar chart of the relative rRNA and mRNA diversity of the microeukaryotes in the four coastal sampling regions: Sweden, Iceland, Greenland, and Norway.

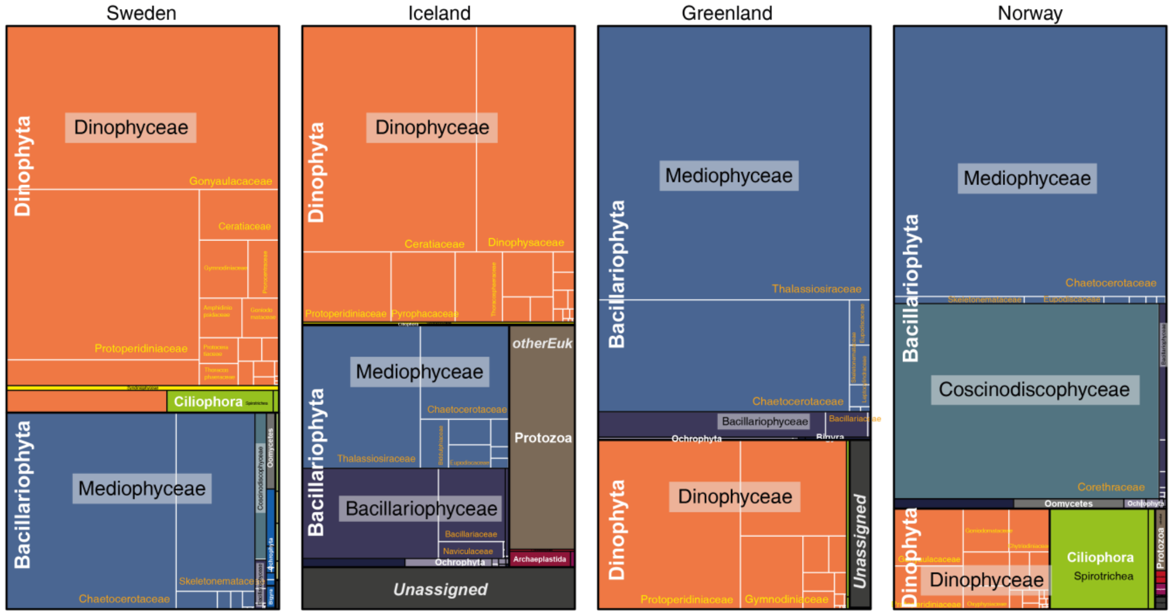

Figure S3: Distribution of taxonomic groups in the four coastal sampling regions: Sweden, Iceland, Greenland, and Norway.

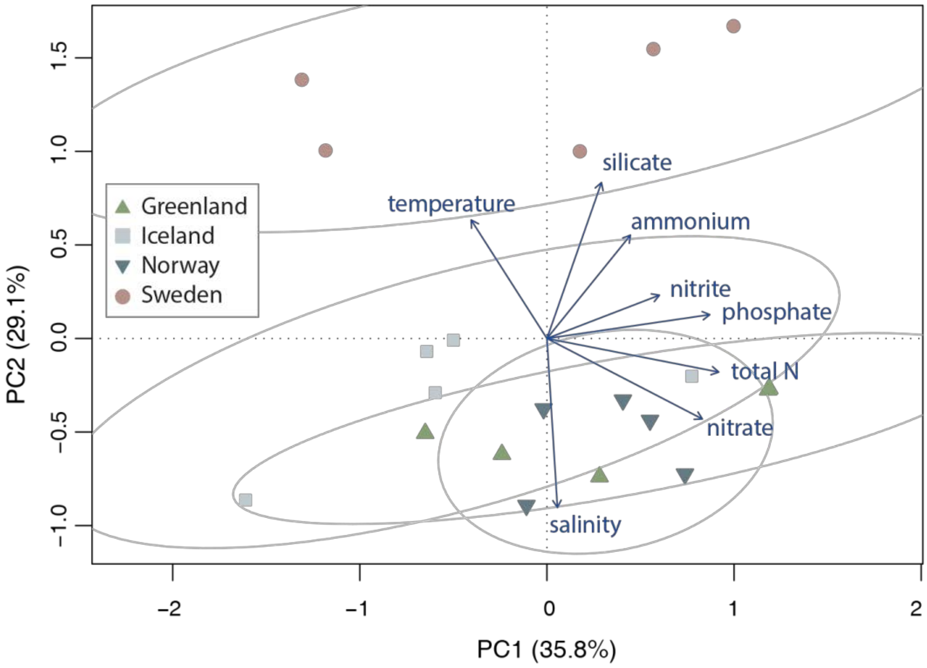

Figure S4: Principle Component analysis (PCA) of Euclidean distances of environmental parameters. Environmental vectors were fitted onto the ordination plot. Ellipses were drawn as 95% confidence limits for the sampling areas for Greenland (triangle up), Iceland (square), Norway (triangle down), and Sweden (dots).

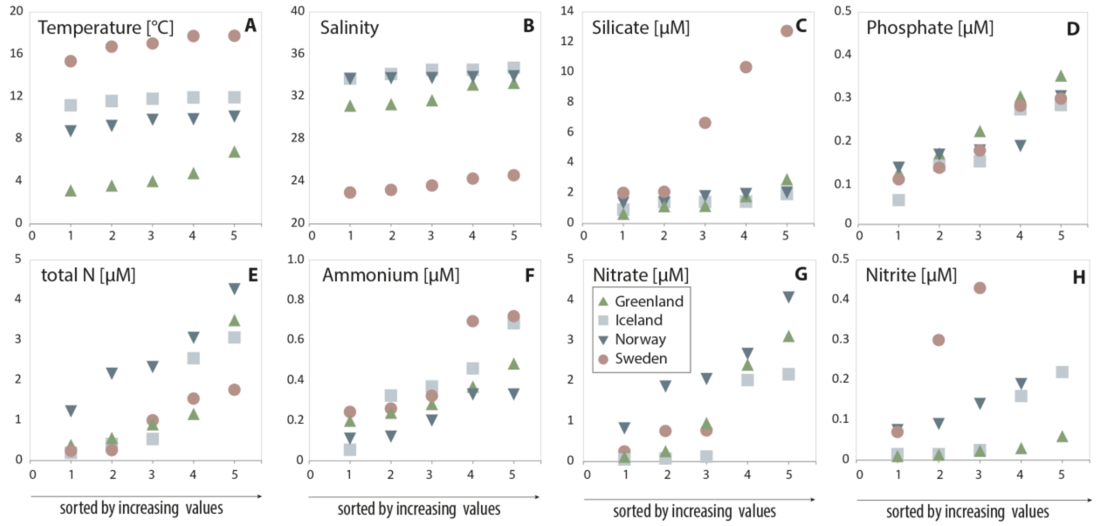

Figure S5: Distribution of environmental parameters (A-H) of the sampling regions in Greenland (triangle up), Iceland (square), Norway (triangle down), and Sweden (dots). The parameters are temperature (A), salinity (B), and inorganic nutrient concentrations (C-H) in the surface layer (mean of 3 m to 30 m). Points represent sampling stations and are sorted by increasing values. For detailed information about the values also see Table S2.

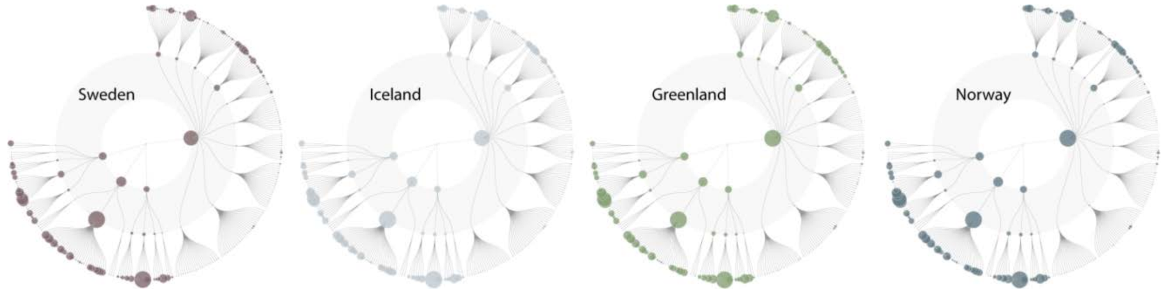

Figure S6: FuncTree2 Plots: All Pfam domains corresponding to Kegg Orthologies (KO) terms (abundance = size) are plotted for each region. The plot shows a very similar distribution in each region and differences in functional diversity are hard to distinguish. Details on the KEGG categories in this plot can be found in Fig. 5.

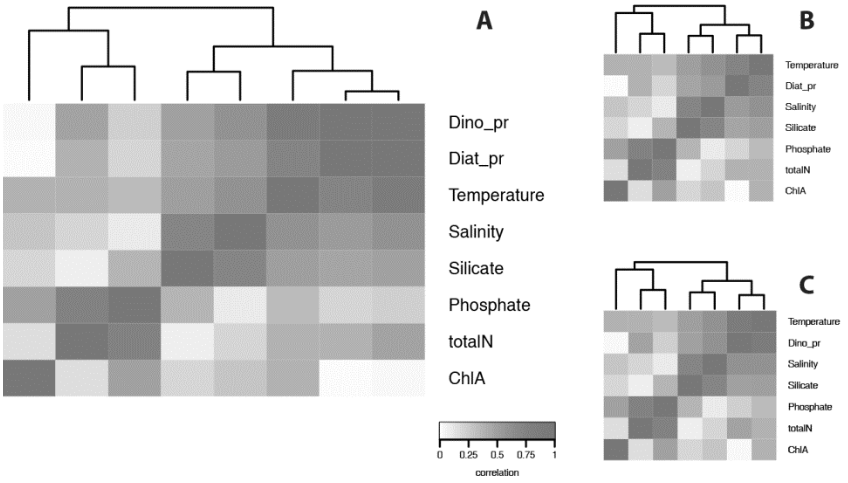

Figure S7: Heat map of correlation between environmental parameters and relative abundance of dinoflagellates and diatoms (A); separate heat maps show the correlation between environmental parameters and diatoms (B) or dinoflagellates (C).

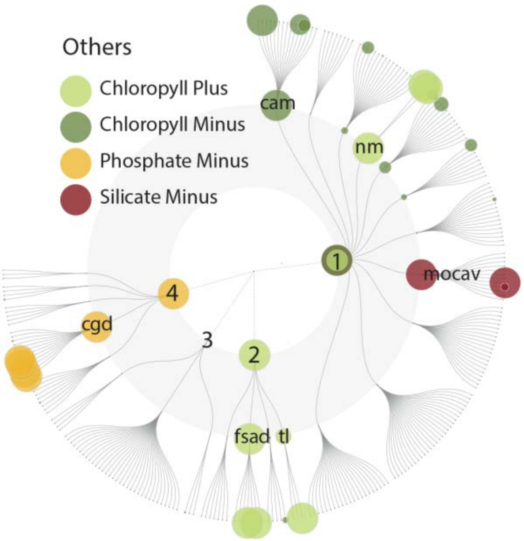

Figure S8: FuncTree 2 Plot: Correlation with Pfam domains and what the variability is most likely related to, including chlorophyll a (positive and negative correlation), phosphate (negative correlations), and silicate (negative correlations). The KEGG biological categories are **metabolism** (1) including carbohydrate metabolism (cam), nucleotide metabolism (nm), and metabolism of cofactors and vitamins (mocav); **genetic information processing** (2) including folding, sorting, and degradation (fsad) and translation (tl); **environmental information processing** (3); and **cellular processes** (4) representing cell growth and death (cgd). More details on the KEGG categories can be found in Fig. 5. Kegg Orthologies (KO) abundance was counted and plotted (size = relative abundance of KOs in each plot).

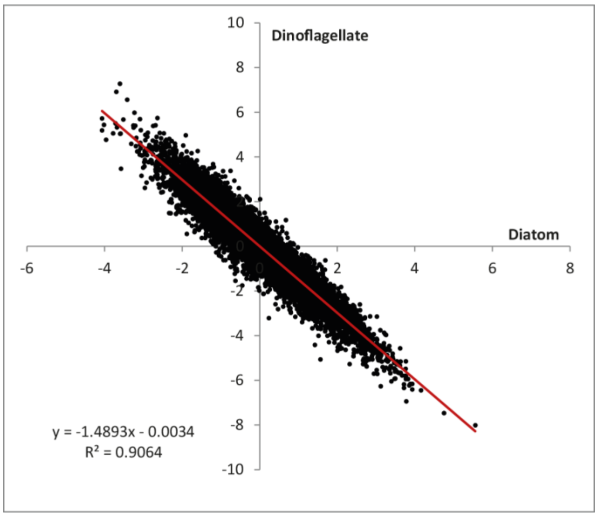

Figure S9: The negative correlation between dinoflagellates and diatom Pfam abundance (from estimates).
