# Supplementary material for: Functional Genomics Differentiate Inherent and Environmentally Influenced Traits in Dinoflagellate and Diatom Communities
Source: Microorganisms. 2020 Apr 15;8(4):567. doi: 10.3390/microorganisms8040567 (PMC7232425; doi:10.3390/microorganisms8040567)
Supplement: Supplementary file 1 [file microorganisms-08-00567-s001.zip › Supplement Tables_2020.04.pdf]

Supplementary tables to

"Functional genomics differentiate inherent and environmentally influenced traits in dinoflagellate and diatom communities"

Table S1 Overview of 454-pyrosequencing reads and number of ASVs after quality processing and removing of singletons, doubletons, potential fungal and metazoan sequences (defined by QIIME) for the microeukaryotic plankton size-fraction (20-50 μm). Raw reads here refer to the reads after quality filtering and before removing singleton and other sequences.

station	raw reads	Microplankton reads	ASVs
G_510	6571	6516	29
G_511	5261	5259	54
G_512	6984	6983	53
G_516	13490	12854	160
G_517	9292	8728	121
I_527	35234	22686	174
I_528	9967	9145	206
I_530	36996	36266	154
I_539	28410	24664	199
I_540	20945	18928	127
N_001	14352	14141	192
N_002	7202	7001	213
N_003	12984	12683	255
N_004	11195	9610	279
N_005	9251	8441	249
S_027	6641	4565	225
S_028	7760	5634	205
S_029	9335	6652	194
S_030	9574	6338	187
S_031	9108	7176	195

Table S2: Details to supplementary figure S5: Environmental parameters concentrations.

	station	Temperature [°C]		station	Salinity [psu]		station	Silicate [µM]		station	Phosphate [µM]
A1	**517**	3.17	B1	**516**	31.17	C1	**516**	0.65	D1	**511**	0.13
A2	**510**	3.63	B2	**517**	31.30	C2	**511**	1.15	D2	**512**	0.17
A3	**516**	4.05	B3	**510**	31.68	C3	**512**	1.17	D3	**516**	0.23
A4	**512**	4.80	B4	**511**	33.16	C4	**510**	1.82	D4	**510**	0.31
A5	**511**	6.83	B5	**512**	33.32	C5	**517**	2.93	D5	**517**	0.35
A1	**527**	11.19	B1	**528**	33.71	C1	**539**	0.915	D1	**539**	0.065
A2	**528**	11.59	B2	**527**	34.15	C2	**528**	1.415	D2	**528**	0.145
A3	**540**	11.81	B3	**541**	34.53	C3	**527**	1.42	D3	**527**	0.155
A4	**541**	11.94	B4	**540**	34.55	C4	**540**	1.45	D4	**540**	0.275
A5	**539**	11.94	B5	**539**	34.72	C5	**541**	1.95	D5	**541**	0.285
A1	**5**	8.74	B1	**1**	33.67	C1	**3**	1.36	D1	**1**	0.14
A2	**3**	9.24	B2	**4**	33.74	C2	**1**	1.39	D2	**2**	0.17
A3	**4**	9.81	B3	**2**	33.76	C3	**2**	1.81	D3	**3**	0.18
A4	**1**	9.88	B4	**3**	33.85	C4	**4**	1.95	D4	**4**	0.19
A5	**2**	10.12	B5	**5**	33.93	C5	**5**	2.06	D5	**5**	0.31
A1	**29**	15.36	B1	**27**	22.95	C1	**27**	2.03	D1	**31**	0.11
A2	**30**	16.72	B2	**30**	23.20	C2	**30**	2.10	D2	**30**	0.14
A3	**27**	17.03	B3	**31**	23.62	C3	**31**	6.66	D3	**27**	0.18
A4	**28**	17.73	B4	**29**	24.27	C4	**28**	10.35	D4	**28**	0.28
A5	**31**	17.76	B5	**28**	24.59	C5	**29**	12.75	D5	**29**	0.30

	station	totalN [µM]		station	Ammonia [µM]		station	Nitrate [µM]		station	Nitrite [µM]
E1	**511**	0.39	F1	**516**	0.20	G1	**516**	0.12	H1	**516**	0.01
E2	**512**	0.56	F2	**511**	0.24	G2	**517**	0.26	H2	**517**	0.01
E3	**510**	0.91	F3	**512**	0.28	G3	**511**	0.96	H3	**512**	0.02
E4	**516**	1.17	F4	**517**	0.37	G4	**510**	2.41	H4	**511**	0.03
E5	**517**	3.50	F5	**510**	0.49	G5	**512**	3.12	H5	**510**	0.06
E1	**539**	0.19	F1	**539**	0.055	G1	**528**	0.05	H1	**528**	0.015
E2	**528**	0.42	F2	**528**	0.325	G2	**527**	0.075	H2	**539**	0.015
E3	**527**	0.54	F3	**540**	0.37	G3	**539**	0.12	H3	**527**	0.025
E4	**540**	2.55	F4	**527**	0.46	G4	**540**	2.02	H4	**540**	0.16
E5	**541**	3.07	F5	**541**	0.685	G5	**541**	2.165	H5	**541**	0.22
E1	**1**	1.23	F1	**5**	0.11	G1	**3**	0.82	H1	**1**	0.08
E2	**3**	2.17	F2	**3**	0.12	G2	**5**	1.86	H2	**5**	0.09
E3	**2**	2.33	F3	**4**	0.20	G3	**1**	2.05	H3	**2**	0.14
E4	**4**	3.06	F4	**1**	0.33	G4	**4**	2.67	H4	**4**	0.19
E5	**5**	4.27	F5	**2**	0.33	G5	**2**	4.07	H5	**3**	
E1	**31**	0.24	F1	**31**	0.24	G1	**30**	0.25	H1	**30**	0.07
E2	**27**	0.26	F2	**27**	0.26	G2	**27**	0.76	H2	**29**	0.30
E3	**28**	1.00	F3	**28**	0.32	G3	**31**	0.77	H3	**28**	0.43
E4	**30**	1.55	F4	**29**	0.70	G4	**28**		H4	**27**	
E5	**29**	1.77	F5	**30**	0.72	G5	**29**		H5	**31**	
